# Job preferences of undergraduate pharmacy students in China: a discrete choice experiment

**DOI:** 10.1186/s12960-021-00626-8

**Published:** 2021-07-06

**Authors:** Ping Liu, Shimeng Liu, Tiantian Gong, Quan Li, Gang Chen, Shunping Li

**Affiliations:** 1 Centre for Health Management and Policy Research, School of Public Health, Cheeloo College of Medicine, Shandong University, Jinan, 250012 China; 2 NHC Key Laboratory of Health Economics and Policy Research (Shandong University), Jinan, 250012 China; 3 Centre for Health Preference Research, Shandong University, Jinan, 250012 China; 4 School of Public Health, Fudan University, Shanghai, 200032 China; 5 NHC Key Laboratory of Health Technology Assessment (Fudan University), Shanghai, 200032 China; 6 Cheeloo College of Medicine, Shandong University, Jinan, 250012 China; 7 Centre for Health Economics, Monash Business School, Monash University, Melbourne, 3145 Australia

**Keywords:** China, Discrete choice experiments, Undergraduate pharmacy students, Job preferences

## Abstract

**Background:**

Pharmacists are a crucial part of the health workforce and play an important role in achieving universal health coverage. In China, pharmaceutical human resources are in short supply, and the distribution is unequal. This study aimed to identify the key job characteristics that influence the job preferences of undergraduate pharmacy students and to elicit the relative importance of different job characteristics to shed light on future policy interventions.

**Methods:**

A discrete choice experiment was conducted to assess the job preferences of undergraduate pharmacy students from 6 provinces in mainland China. A face-to-face interview was used to collect data. Conditional logit and mixed logit models were used to analyse data, and the final model was chosen according to the model fit statistics. A series of policy simulations was also conducted.

**Results:**

In total, 581 respondents completed the questionnaire, and 500 respondents who passed the internal consistency test were analysed. All attributes were statistically significant except for open management. Monthly income and work location were most important to respondents, followed by work unit (which refers to the nature of the workplace) and years to promotion. There was preference heterogeneity among respondents, e.g., male students preferred open management, and female students preferred jobs in public institutions. Furthermore, students with an urban background or from a single-child family placed higher value on a job in the city compared to their counterparts.

**Conclusion:**

The heterogeneity of attributes showed the complexity of job preferences. Both monetary and nonmonetary job characteristics significantly influenced the job preferences of pharmacy students in China. A more effective policy intervention to attract graduates to work in rural areas should consider both incentives on the job itself and the background of pharmacy school graduates.

**Supplementary Information:**

The online version contains supplementary material available at 10.1186/s12960-021-00626-8.

## Background

Overuse and misuse of drugs seriously threaten the life and health of patients, leading to the waste of scarce resources and widespread health hazards [1]. The World Health Organization (WHO) estimated that more than half of prescription drugs were improperly dispensed or marketed, and half of the patients used them incorrectly [1]. Pharmacists are an important part of the health workforce and an important force in achieving universal health coverage [2]. In the people-centred integrated care model, pharmacists play a vital role in providing drug knowledge, guiding rational clinical drug use, ensuring drug quality and providing pharmaceutical services [3–5].

Many countries worldwide are facing a shortage of pharmaceutical human resources and an imbalance of internal distribution, a situation that is also occurring in China [6, 7]. Although the number of pharmacists in China has increased in recent years, the ratio of doctors, nurses and pharmacists is unbalanced. According to statistics, in 2018, the ratio of doctors, nurses and pharmacists in China was 7.71/8.76/1 [8]. Furthermore, the distribution of pharmacists in rural and urban areas is unequal. In 2018, the number of pharmacists per 10,000 people in urban and rural areas was 4.08 and 2.80, respectively [8, 9]. In addition, pharmacists in China are faced with the problem of a low education level [10, 11]. Less than one-third of pharmacists have a bachelor’s degree, and only 3.4% of them have a graduate degree [8].

One study found that pharmacists had the lowest job satisfaction compared with doctors, nurses and administrators [12]. The factors influencing pharmacists’ job satisfaction are complex and mainly include salary, working environment, leadership and management style, promotion and training opportunities [12–14]. Pharmacy students are regarded as potential pharmacists. To promote a better allocation of pharmaceutical human resources and the development of pharmaceutical care, it is important to understand the job preferences of pharmacy students [15, 16].

Discrete choice experiments (DCEs) are a stated preference technique that has been widely used in the health workforce to facilitate effective policy intervention for recruitment and retention in both developed and developing countries [17]. In China, DCEs are increasingly being used to explore the job preferences of health professionals, including doctors, nurses and public health personnel in primary health institutions [18–22], along with medical students, nursing students and health management students [23–25]. However, no study has been conducted with pharmacy students in China. Globally, there have been two DCE studies exploring the job preferences of pharmacy students. The first study identified six attributes and surveyed 283 students in total from the United States and Canada [26]. This study found significant differences between states and provinces in the job preferences of pharmacy students. The second was from Uganda [27], in which pharmacy students, as one of four types of trainee health professionals, were surveyed. This study showed that pharmacy students placed high value on the opportunity to operate a private pharmacy in addition to working at a public health facility, referred to as dual practice or moonlighting.

This study aimed to investigate the relative importance of attributes influencing the job preferences of undergraduate pharmacy students in mainland China. The results from this study provide important information for policymakers to design and improve employment policies to improve the rational distribution of pharmaceutical human resources in China.

## Methods

A DCE, which is based on random utility theory, is a quantitative method that can be used to measure respondents’ preferences and has been widely used in economics, marketing and psychology [28]. In a DCE, respondents are asked to choose their preferred option from hypothetical alternatives that contained one or more combinations of attributes and levels [29, 30]. In this study, a DCE was designed and analysed following the user guide published by the WHO [31] and the checklist published by the International Society for Pharmacoeconomics and Outcomes Research (ISPOR)[32].

### Attributes and levels

Developing attributes and levels is a key step in DCEs [33, 34]. Both a literature review and qualitative studies were conducted to ensure that the attributes and levels included were the most meaningful for respondents. First, we identified 9 attributes from a literature review, including monthly income, work environment, work location, *bianzhi* (which refers to the established posts and can be loosely regarded as state administrative staffing), workload, training opportunity, career development opportunity, management style, and welfare [23–27]. Next, we consulted 3 clinical pharmacists, who provide clinical pharmacy services in hospitals and have rich work and research experience. Based on their suggestions on the importance and feasibility of attributes and levels, we removed two attributes (the workload and bianzhi), added in one new attribute (the years to the promotion), and adjusted the levels of monthly income attribute. In addition, we also consulted 3 researchers with experience in conducting DCEs on human resources for health, and they suggested that two attributes (welfare and career development opportunities) could be removed. Then, we conducted 6 in-depth interviews with students from Shandong University of Traditional Chinese Medicine and Weifang Medical University. Based on their feedback, the work environment attribute was removed because most students thought it was not important. Finally, a focus group discussion (with 7 undergraduate pharmacy students) from Shandong University was conducted. Participants were asked to discuss the remaining 5 attributes and levels until they reached a consensus on the final version of the attributes and levels. They were also provided with an opportunity to add in additional attributes they considered important but not on the list. One additional attribute (and its corresponding levels), i.e., the work unit (which refers to the nature of the workplace), was added at the end. The final 6 attributes and levels are shown in Table 1.

**Table 1:** Attributes and attributes levels for DCE choice questions

Attributes	Definition	Attributes levels
Monthly income	Monthly income including salary, bonus and welfare benefits	3000 yuan
		6000 yuan
		9000 yuan
Work location	Location refers to working in different regions	Township or village
		County
		City
Work unit	Work unit refers to the nature of your workplace. Public institutions such as hospitals, food and drug administration, etc.; foreign-funded enterprises include pure foreign-capital and sino-foreign joint ventures; State-owned enterprises include state-owned enterprises, central enterprises and so on; Private enterprises such as private medical enterprises	Public institutions
		Foreign-funded enterprises
		State-owned enterprises
		Private enterprises
Management style	Management style refers to the understanding, support and adoption of the work and Suggestions of the employees by the company or leadership, as well as the degree of freedom of the employees in the work	Semi-open management
		Open management
Training opportunity	Training opportunity refers to during the employment period to accept the company to provide a variety of skills training opportunity	Insufficient
		Average
		Sufficient
Years to promotion	Years to promotion refers to the number of years required for promotion	5 years
		2 years

According to the Organisation for Economic Co-operation and Development (OECD) data, the average annual exchange rate between US$ and CNY in 2017 was 1$ = 6.759 yuan

### DCE design

Of the 6 attributes, 2 are two-level, 3 are three-level, and 1 is four-level. A full factorial design will produce 432 (= 2^2^ × 3^3^ × 4^1^) hypothetical scenarios and 93,096 (=  (432 × 431)/2) pairwise choice tasks. A D-efficient design was used to generate 24 manageable choice sets using DCE design software Ngene 1.1.2 (Choice-Metrics, Sydney, Australia) [35]. To reduce the response burden of respondents, the 24 choice sets were further divided into two blocks. An opt-out was included in the second-stage question after each DCE task to allow for unconditional choices [36]. In the first step, respondents were asked to choose the job they preferred from two hypothetical jobs, and in the second step, they were asked to answer whether they would take the job if it were available in real life. An example of a DCE choice set is shown in Table 2. To check for internal consistency, one choice set was duplicated. Hence, in total, every respondent answered 13 DCE questions. Respondents who failed the consistency test were excluded from the main analyses following the previous literature [37, 38].

**Table 2:** An example of DCE choice set

Attributes	Job 1	Job 2
Monthly income	6000 yuan	9000 yuan
Work location	City	County
Work unit	Foreign-funded enterprises	Public institutions
Management style	Open management	Semi-open management
Training opportunity	Average	Sufficient
Years to promotion	5 years	2 years
Which job do you prefer?	▢	▢
Would you choose this job in real life?	Yes	No

### Sampling

**Fig. 1:**
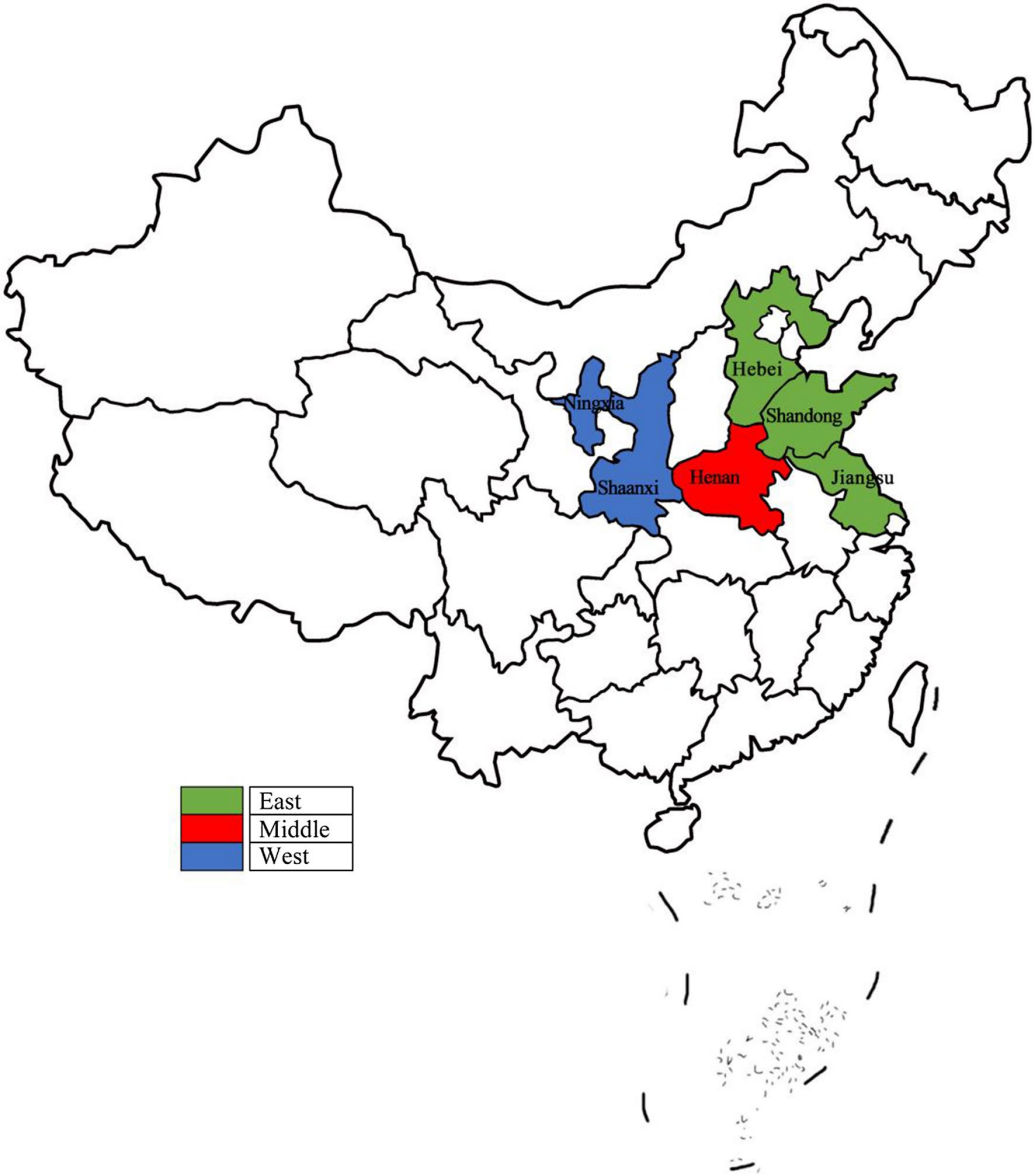
The provinces selected in China

Final-year undergraduate pharmacy students were chosen as the targeting respondents in this study given that the questions were highly relevant for these students, as they would be on the job market soon. A multistage cluster sampling design was used. Firstly, we selected 6 provinces to represent eastern (Hebei, Shandong and Jiangsu), middle (Henan) and western (Shaanxi and Ningxia) China according to the level of economic development and the geographical location. Secondly, a representative university offering pharmacy degree was selected in each province according to the type of universities, including 3 general universities (Shandong University, Henan University, Xi’an Jiaotong University) and 3 medical universities (Hebei Medical University, China Pharmaceutical University, Ningxia Medical University). Finally, 1 to 2 graduation classes of students majoring in pharmacy were randomly selected from each school according to the sample size requirements of each region. The sampling map is shown in Fig. 1.

The sample size for DCE is not straightforward and depends on many factors, such as the number of attributes and levels, the number of choice tasks, and the accuracy of expected results [39]. A rule of thumb based on the number of attributes and levels is commonly used to estimate the sample size [40]. Accordingly, it was calculated that the sample size required for this study should be more than 83 respondents (500*4/ (2*12) = 83). Combined with the sample size requirement of de Bekker-Grob [41] (i.e., how to calculate the sample size for healthcare-related DCE studies) and the minimum sample size requirement proposed by Pearmain et al. [42], the sample size should be no less than 100 respondents. A review of domestic studies on discrete choice experiments in human resources for health found that about 70% of the studies had a sample size of less than 500 people. Therefore, we decided that the sample size in eastern, middle and western China should be more than 100, and the total sample size should be no less than 500.

### Data collection

Before the formal survey, we conducted a pre-test among final-year undergraduate pharmacy students at Shandong University. In the pre-test, we tested whether the attributes and corresponding levels were reasonable and easy to understand. Minor adjustments were made based on the pre-test. Finally, a face-to-face anonymous survey was conducted from April to July 2017.

The questionnaire consists of two parts: section one collects personal background information, and section two is the DCE. Prior to the survey, we obtained oral informed consent from all respondents. This study was approved by the Ethics Review Board of the School of Preventive Medicine, Shandong University (Reference No. 20170301).

### Data analysis

The DCE data were analysed using a conditional logit (CL) model (which assumes a homogeneous preference among respondents) and a mixed logit (MIXL) model (which allows for potential preference heterogeneity). Based on the random utility framework, the utility function can be expressed as:

$$U_{{njt}} = V_{{njt}} + \varepsilon _{{njt}} = \beta ^{\prime}_{n} X_{{njt}} + \varepsilon _{{njt}} ,$$

where *U*_*njt*_ refers to the utility obtained by respondents *n* by choosing alternatives *j* in choice scenario *t*. *U*_*njt*_ consists of the observable component *V*_*njt*_ and the unobservable component *ε*_*njt*_. The observable component is equal to the attribute level vector *X*_*njt*_ multiplied by the coefficient vector $\beta ^{\prime}_{n}$_**,**_ and the unobservable component is a random error term. Except for monthly income, which was treated as a continuous variable for the calculation of willingness to pay (WTP), all other attributes were coded as dummy variables [43]. WTP is calculated by $- \frac{{\mathop \beta \nolimits_{{\text{q}}} }}{{\mathop \beta \nolimits_{m} }}$, where *β*_*m*_ is the monthly income coefficient and *β*_*q*_ is the coefficient for attribute levels *q* [44]. In this context, the WTP shows the relative monetary value that pharmacy students place on different job characteristics, which will facilitate our understanding of the relative importance of nonmonetary attributes in DCEs.

In MIXL, coefficients of attribute levels are usually assumed to follow a normal distribution (described based on a mean coefficient and a standard deviation). The mean coefficients reflect the relative preference weights, and the standard deviation reflects the extent of preference heterogeneity [45, 46]. The choice between CL and MIXL was guided by model fit statistics, including log-likelihood ratio tests, Akaike information criterion (AIC) and Bayesian information criterion (BIC). The distance between the best and worst preference weights within each attribute can be used to compare the relative importance of different attributes to the respondents. Subgroup analyses were also conducted. After estimating the regression coefficients, a series of policy simulations were conducted to predict the probability of job choices given the changes in attributes levels, the results of which would be of interest to policymakers. Descriptive statistics were also presented. All statistical analyses were conducted using Stata software version 15.1 (StataCorp LP, College Station, TX, USA).

## Results

A total of 617 final-year undergraduate pharmacy students were surveyed; among them, 36 (5.8%) students did not complete the questionnaire. The sample that completed the questionnaire was representative in terms of age, gender, birthplace and career intention. As shown in Table 3, the mean age of the remaining 581 (94.2%) students was 22.3 years and over 70% were female students, which is comparable to a previous study and reflects the age and gender composition ratio of undergraduate pharmacy students [47]. Nearly two-thirds had siblings, and more than half were born in townships or villages. For career planning, more than 80% of them were pursuing a career related to pharmacy or were continuing to study pharmacy. Most respondents wanted to work in public institutions and foreign pharmaceutical companies, which was consistent with a study on the career intention of undergraduate pharmacy students from China [48]. Of 581 respondents who returned the questionnaire, 81 (13.9%) consequently failed the consistency test. There were no significant differences in respondent characteristics between those who failed the test and those who were included in the main analyses. CL was used for sensitivity analysis. There were also no significant differences in preferences between the full sample, the forced choice sample and the respondents who passed the consistency test (see Additional file 1: Tables S1–S3 for details). Given that the use of opt-outs is more realistic, we analysed the data containing opt-outs, and 500 respondents were included in the main analyses.

**Table 3:** Socio-demographic characteristics of the respondents

Characteristics of respondents	All (*N* = 581)	Respondents who passed the consistency test (*N* = 500)	Respondents who failed the consistency test (*N* = 81)	*χ*^2^	*P* value
Age (years), Mean, (± SD)	22.3 (± 1.0)	22.3 (± 1.0)	22.4 (± 1.3)		
Gender					
Male	169 (29.1%)	142 (28.4%)	27 (33.3%)	0.823	0.364
Female	412 (70.9%)	358 (71.6%)	54 (66.7%)		
Birthplace					
City	156 (26.9%)	132 (26.4%)	24 (29.6%)	0.640	0.726
County	109 (18.8%)	96 (19.2%)	13 (16.0%)		
Township or village	316 (54.4%)	272 (54.4%)	44 (54.3%)		
Single child					
Yes	216 (37.2%)	185 (37.0%)	31 (38.3%)	0.048	0.826
No	365 (62.8%)	315 (63.0%)	50 (61.7%)		
Monthly consumption level (yuan)					
< 800	90 (15.5%)	75 (15.0%)	15 (18.5%)	3.409	0.333
800–1500	377 (64.9%)	328 (65.6%)	49 (60.5%)		
1501–2500	100 (17.2%)	87 (17.4%)	13 (16.0%)		
> 2500	14 (2.4%)	10 (2.0%)	4 (4.9%)		
Annual family income (yuan)					
< 30,000	138 (23.8%)	112 (22.4%)	26 (32.1%)	8.253	0.083
30,000–50,000	170 (29.3%)	151 (30.2%)	19 (23.5%)		
50,001–70,000	104 (17.9%)	87 (17.4%)	17 (21.0%)		
70,001–90,000	69 (11.9%)	65 (13.0%)	4 (4.9%)		
> 90,000	100 (17.2%)	85 (17.0%)	15 (18.5%)		
Career planning					
Engaged in pharmaceutical work	138 (42.2%)	217 (43.4%)	28 (34.6%)	6.313	0.097
Continue to study pharmacy	170 (44.1%)	221 (44.2%)	35 (43.2%)		
Continue to study in other fields	104 (8.8%)	39 (7.8%)	12 (14.8%)		
Others	69 (5.0%)	23 (4.6%)	6 (7.4%)		
Work unit					
Public institutions	222 (38.2%)	186 (37.2%)	36 (44.4%)	2.699	0.609
Foreign-funded enterprises	209 (36.0%)	185 (37.0%)	24 (29.6%)		
State-owned enterprises	109 (18.8%)	93 (18.6%)	16 (19.8%)		
Private enterprises	23 (4.0%)	21 (4.2%)	2 (3.7%)		
Others	18 (3.1%)	15 (3.0%)	3 (2.5%)		

### Ranking results

**Fig. 2:**
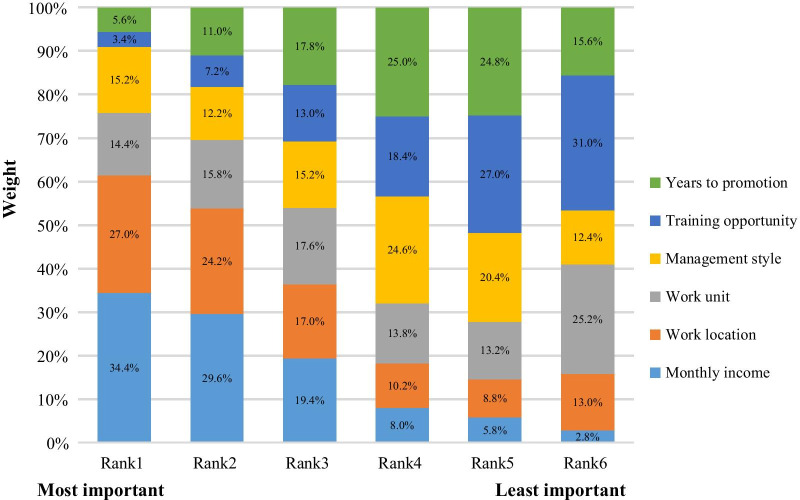
The relative importance of attributes

Among the attributes, monthly income was the most important attribute to the respondents, followed by work location, whereas training opportunities and years to promotion were less important. For example, among 500 respondents, more than one-third regarded monthly income as the most important attribute, and nearly one-third considered training opportunities to be the least important attribute. The relative importance of attributes from analysing the ranking question is shown in Fig. 2.

### DCE results

Both the CL and MIXL models were initially applied for data analysis. Based on information criteria (i.e., the log-likelihood ratio test, AIC and BIC), the MIXL estimates were preferable and are reported in Table 3, while the CL estimates can be found in Additional file 1: Table S1. According to the MIXL estimates, all attribute levels were statistically significant except management style. On average, respondents preferred jobs in the city, with high monthly income, to work in public institutions, with sufficient training opportunities and fewer promotion years. The statistically significant SDs indicate that preference heterogeneity existed among respondents in all significant attributes. See Table 4 for details.

**Table 4:** Mixed logit model results

Attributes	*β*	SE	SD	SE	WTP	95% CI	
Opt-out	5.811***	0.257	2.402***	0.167	–	–	–
Monthly income	0.000654***	0.000025	0.000202***	0.000019	–	–	–
Work location (ref. Township or village)							
County	0.890***	0.084	0.772***	0.126	1360.7	1117.9	1611.8
City	1.890***	0.105	1.399***	0.108	2888.8	2587.3	3200.8
Work unit (ref. Private enterprises)							
State-owned enterprises	0.777***	0.096	0.789***	0.138	1186.8	907.1	1471.4
Foreign-funded enterprises	0.930***	0.091	0.508**	0.178	1421.2	1164.5	1682.5
Public institutions	0.976***	0.098	0.888***	0.127	1490.9	1208.1	1782.3
Management style (ref. Semi-open management)							
Open management	0.052	0.055	0.075	0.160	80.2	-84.3	247.5
Training opportunity (ref. Insufficient)							
Average	0.360***	0.075	0.211	0.258	550.0	324.9	783.6
Sufficient	0.763***	0.078	0.556***	0.141	1166.0	936.9	1404.7
Years to promotion (ref.: 5 years)							
2 years	0.971***	0.066	0.700***	0.095	1484.2	1285.1	1688.1
AIC	8216.947						
BIC	8388.505						
Log likelihood	− 4086.473						
Respondents, n	500						
Observations, *n*	18,000						

*β* coefficient, *SE *standard error, *SD *standard deviation, ref-reference, *AIC *Akaike information criterion, *BIC *Bayesian information criterion, – none or not applicable, *95% CI* 95% Confidence Interval

*P < 0.05, **P < 0.01, ***P < 0.001

### Willingness to pay

We found a clear preference among respondents regarding work location. Respondents were willing to pay 2889 yuan (US$427) for a job in the city over a job in the county or village. If the training opportunities increased from insufficient to sufficient, respondents were willing to pay 1166 yuan (US$172) per month. Furthermore, they were willing to pay almost the same money to work in public institutions or to shorten the promotion years from 5 to 2 years.

### Policy simulation

**Fig. 3:**
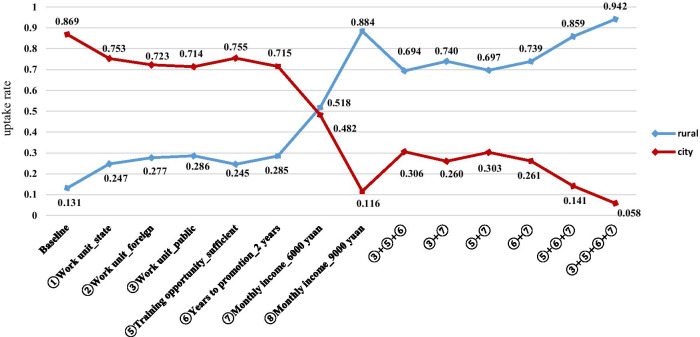
Policy simulation analysis of taking a job with specific attributes (Baseline: Monthly income_3000 yuan, Work unit_private, Training opportunity_insufficient, Years to promotion_5 years)

The changes in the probability of taking a job in rural areas (township or village) are shown in Fig. 3. We set the monthly income of 3000 yuan (US$444), private enterprises, insufficient training opportunities, and 5 years to promotion as the baseline scenario. The initial probability of taking a job in the city was 0.869, whereas the probability of taking a job in rural areas was only 0.131. In the policy simulation analyses, holding all else the same, when the monthly income increased from 3000 yuan (US$444) to 9000 yuan (US$1332) and other factors remained constant, the probability of working in rural areas increased to 0.884. The effect of a single noneconomic incentive for taking a rural job was not very obvious. For the multiple incentives, the combination “③ + ⑤ + ⑥ + ⑦” was the most effective, and the probability of choosing a job in rural areas was 0.942.

### Subgroup analysis

The detailed results of the subgroup analyses are shown in Additional file 1: Tables S4–S8, and the WTP results for the subgroups are shown in Table 5. We found that the respondents who were only children or were from the city were more likely to work in the city (based on the calculated WTP values, 95% CI). In addition, female students preferred to work in the city compared with male students. Open management was statistically significant for males but not for females, and students in eastern and central China placed more value on public institutions. Regarding training opportunities, students in the east valued them more, as they were willing to pay 1306 yuan (US$193) to obtain sufficient training opportunities. Students in the middle of the country paid more attention to years to promotion and were willing to pay 1683 yuan (US$249) to reduce the years to promotion from 5 to 2 years.

**Table 5:** Willingness to pay for subgroups; 95%CI

Attributes levels	City	Country or Rural	Family income ≤ 50,000	Family income > 50,000	Male	Female	Single child	Non- single child	East	Middle	West
Work location (ref.Township or village)											
County	1231.8	1399.4	1298.5	1400.8	1220.8	1411.8	1428.0	1297.8	1073.5	1771.4	1666.0
City	4392.1	2312.4	2256.2	3469.6	2566.7	3044.7	3654.9	2382.5	2675.5	3006.5	3332.2
Work unit (ref.Private enterprises)											
State-owned enterprises	1577.0	1041.8	1061.3	1320.3	1062.3	1224.4	1203.9	1139.9	1100.5	821.1	1683.5
Foreign-funded enterprises	1432.4	1395.6	1287.9	1554.8	1336.0	1432.1	1386.7	1385.0	1477.0	1015.0	1680.6
Public institutions	1465.6	1535.4	1420.9	1573.5	1103.6	1652.8	1315.7	1597.6	1477.2	1433.4	1266.5
Management style (ref.Semi-open management)											
Open management	–	–	–	–	526.0	− 81.0	–	–	–	–	–
Training opportunity (ref.Insufficient)											
Average	1009.8	406.5	278.0	847.4	580.2	546.7	965.9	318.9	645.9	347.3	330.7
Sufficient	1380.1	1095.3	1116.6	1208.1	1262.3	1150.2	1219.9	1136.4	1306.2	564.2	1236.6
Years to promotion (ref.5 years)											
2 years	1225.0	1416.4	1438.6	1548.8	1400.9	1528.6	1373.4	1538.9	1319.4	1682.7	880.6

According to the Organisation for Economic Co-operation and Development (OECD) data, the average annual exchange rate between US$ and CNY in 2017 was 1$ = 6.759 yuan

*ref* reference, – not applicable

## Discussion

To date, globally, there are very limited DCE studies on the job preferences of pharmacy students. This is the first study to investigate the job preferences of undergraduate pharmacy students in China. Six job characteristics were identified from a literature review and qualitative studies, and we found that all of them were statistically significant except management style.

Work location was the most important nonmonetary attribute to pharmacy students. The lower economic development status in rural areas hinders the attraction of human resources for health. Among respondents, we can see that students who were the only child in the family, female, with an urban background, or from a wealthier family placed stronger value on work location than their counterparts. This finding is consistent with a previous study in the United States, in which the authors found that female students preferred to work in the city and that they required a larger financial incentive to move to rural areas [26]. When designing a more effective policy implementation to attract pharmacy students to work in rural areas, it would be important to take into account their backgrounds, e.g., male students who are not single children in their families and are originally from rural areas would be more likely to take a job in a rural area after graduation.

Compared with male students, female students preferred work in public institutions. The concepts of traditional Chinese families generally believe that girls are better suited to stable jobs such as pharmacists, nurses and teachers, while boys are more suitable for science and engineering majors. Additionally, male students may be more likely to choose a more challenging job offered by a foreign company. This finding is also reflected in another survey conducted in China, which found that nearly half of undergraduate pharmacy students chose sales positions in enterprises, and most of them were male students [49].

The monetary attribute (i.e., monthly income) remains an important factor for job choice among pharmacy students. Similar to a finding in a Canadian study [50], monthly income was perceived to be the most important factor in the ranking analysis. Meanwhile, although some nonmonetary attributes (e.g., training opportunities and years to promotion) significantly influenced pharmacy students’ job preferences, the strengths of their influences were weak. In rural or less developed areas where it is infeasible to purely rely on monetary incentives, a combination of both monetary and nonmonetary interventions could be a more feasible and equally effective approach. When a job has a monthly income of 6000 yuan (US$888), sufficient training opportunities and 2-year promotion, the probability of taking a rural job can be as high as 86%, which has almost the same effect as increasing the monthly income to 9000 yuan (US$1332).

Open management had no statistical significance for the respondents. This means that when respondents trade off the 6 attributes, open management is not very important. However, in the results of the gender subgroup analysis, we found that males had a positive preference for open management. Therefore, we speculated that the reason why the open management coefficient was not significant was that more than 70% of respondents were female students in our study. However, this finding reflected the gender composition of pharmaceutical human resources. Statistically, the proportions of male and female pharmacists in 2018 were 33% and 67%, respectively [8]. Therefore, in the future, how females understand management style and what kind of management style they prefer can be further clarified.

In China, pharmacy students receive less attention than medical students and nursing students [51], and their job preferences could vary. This would be helpful for policymakers when implementing policies between different targeted populations. Hence, we roughly compared their preferences based on previous findings of DCE studies in China [24, 25]. Although there could be some differences in attributes and levels, some results were comparable. For example, three types of students all placed a high value on monthly income. The difference was that work location was the most important for pharmacy students and medical students but was the least important for nursing students. Nursing students valued workload more than anything else. Therefore, students from different majors could focus on different attributes. The characteristics of the major should be taken into account when formulating health workforce policies.

This is the first DCE study in China that focuses on pharmacy students’ job preferences. In addition, we were able to recruit respondents from 6 provinces in China that covered different geographic locations and development stages. This recruitment increased the representation of the findings presented in this study. However, there were also some limitations. First, we did not select provinces and schools by completely random sampling due to the limitation of time and funds, but we comprehensively considered the development level of regions and pharmacy subject and the convenience of the survey. And we are not able to calculate a post-stratification weight for this study given there is no detailed national statistics for the final-year undergraduate pharmacy students in China, which may affect the generalization of the results. Second, we only considered the 6 most important attributes in this DCE, and there could be other factors that influenced pharmacy students’ job choice that were omitted. This was a trade-off between the extensiveness of information to be presented versus the cognitive burden of the respondents. Last, as with all DCE studies, the external validity of the results has not yet been explored. Given the different health systems implemented in different countries, the extent to which the results from Chinese pharmacist students can apply to other countries is unknown.

## Conclusion

As China’s population ages and the prevalence of chronic diseases increases, there is a growing demand for pharmaceutical care. Undergraduate pharmacy students are potential pharmaceutical human resources. This study found that monthly income and working location were the top two important characteristics for students’ job preferences. Preference heterogeneity was observed in that students’ background also influenced their job preferences. The findings from this study will be relevant to policymakers to design a more effective recruitment plan for pharmacy students in China.

## Supplementary Information

**Additional file 1: Tables S1–S3.** Results from a conditional logit model for final-year undergraduate pharmacy students who passed the consistency test (n = 500)/forced choice (n = 533)/full sample (n = 581).    **Tables S4–S8**. Detailed results of the subgroup analysis of birthplace, annual family income, sex, single child status and universities.

## Data Availability

The data used and/or analysed during the study are available from the corresponding author upon reasonable request.
