# Supplementary material for: Job preferences of undergraduate pharmacy students in China: a discrete choice experiment
Source: Hum Resour Health. 2021 Jul 6;19:79. doi: 10.1186/s12960-021-00626-8 (PMC8259344; doi:10.1186/s12960-021-00626-8)
Supplement: Supplementary file 1 — Additional file 1: Tables S1–S3. Results from a conditional logit model for final-year undergraduate pharmacy students who passed the consistency test (n = 500)/forced choice (n = 533)/full sample (n = 581). Tables S4–S8. Detailed results of the subgroup analysis of birthplace, annual family income, sex, single child status and universities. [file 12960_2021_626_MOESM1_ESM.docx]

**SUPPLEMENTARY MATERIAL**

**Supplementary Table 1.** Conditional logit estimates-Respondents who passed the consistency test (n=500).

| **Attributes** | **β** | **SE** |
| --- | --- | --- |
| **Opt-out** | 4.204^***^ | 0.161 |
| **Monthly income** | 0.000423^***^ | 0. 000013 |
| **Work location (ref.Township or village)** |  |  |
| County | 0.586^***^ | 0.060 |
| City | 1.250^***^ | 0.072 |
| **Work unit (ref.Private enterprises)** |  |  |
| State-owned enterprises | 0.532^***^ | 0.067 |
| Foreign-funded enterprises | 0.586^***^ | 0.062 |
| Public institutions | 0.645^***^ | 0.064 |
| **Management style (ref.Semi-open management)** |  |  |
| Open management | 0.079^*^ | 0.037 |
| **Training opportunity (ref.Insufficient)** |  |  |
| Average | 0.302^***^ | 0.055 |
| Sufficient | 0.546^***^ | 0.054 |
| **Years to promotion (ref.: 5 years)** |  |  |
| 2 years | 0.718^***^ | 0.045 |
| **AIC** | 9622.832 | |
| **BIC** | 9708.612 | |
| **Log likelihood** | -4800.416 | |
| **Respondents, n** | 500 | |
| **Observations, n** | 18000 | |

Note:β-coefficient, SE-standard error, ref-reference, AIC-Akaike information criterion, BIC-Bayesian information criterion, 95% CI = 95% Confidence Interval, ^*^p < 0.05, ^**^p < 0.01, ^***^p < 0.001.

**Supplementary Table 2.** Conditional logit estimates-Forced choice (n=533).

| **Attributes** | **β** | **SE** |
| --- | --- | --- |
| **Work location (ref.Township or village)** |  |  |
| County | 0.556^***^ | 0.051 |
| City | 0.993^***^ | 0.065 |
| **Work unit (ref.Private enterprises)** |  |  |
| State-owned enterprises | 0.301^***^ | 0.061 |
| Foreign-funded enterprises | 0.506^***^ | 0.058 |
| Public institutions | 0.393^***^ | 0.064 |
| **Management style (ref.Semi-open management)** |  |  |
| Open management | 0.075^*^ | 0.032 |
| **Training opportunity (ref.Insufficient)** |  |  |
| Average | 0.152^**^ | 0.049 |
| Sufficient | 0.422^***^ | 0.051 |
| **Years to promotion (ref.5years)** |  |  |
| 2years | 0.637^***^ | 0.040 |
| **Monthly income** | 0.000369 ^***^ | 0.000013 |
| **AIC** | 5705.658 | |
| **BIC** | 5780.224 | |
| **Log likelihood** | -2842.829 | |
| **Respondents, n** | 533 | |
| **Observations, n** | 12792 | |

Note:β-coefficient, SE-standard error, ref-reference, AIC-Akaike information criterion, BIC-Bayesian information criterion, 95% CI = 95% Confidence Interval, ^*^p < 0.05, ^**^p < 0.01, ^***^p < 0.001

**Supplementary Table 3.** Conditional logit estimates-Full sample (n=581).

| **Attributes** | **β** | **SE** |
| --- | --- | --- |
| **Work location (ref.Township or village)** |  |  |
| County | 0.469^***^ | 0.052 |
| City | 1.031^***^ | 0.066 |
| **Work unit (ref.Private enterprises)** |  |  |
| State-owned enterprises | 0.542^***^ | 0.060 |
| Foreign-funded enterprises | 0.540^***^ | 0.056 |
| Public institutions | 0.608^***^ | 0.061 |
| **Management style (ref.Semi-open management)** |  |  |
| Open management | 0.060 | 0.034 |
| **Training opportunity (ref.Insufficient)** |  |  |
| Average | 0.245^***^ | 0.049 |
| Sufficient | 0.514^***^ | 0.047 |
| **Years to promotion (ref.5years)** |  |  |
| 2years | 0.638^***^ | 0.040 |
| **Monthly income** | 0.000388^***^ | 0.000012 |
| **Opt-out** | 3.829^***^ | 0.147 |
| **AIC** | 11746.850 | |
| **BIC** | 11834.280 | |
| **Log likelihood** | -5862.427 | |
| **Respondents, n** | 581 | |
| **Observations, n** | 20916 | |

Note:β-coefficient, SE-standard error, ref-reference, AIC-Akaike information criterion, BIC-Bayesian information criterion, 95% CI = 95% Confidence Interval, ^*^p < 0.05, ^**^p < 0.01, ^***^p < 0.001

**Supplementary Table 4.** Sub-group analysis: Birthplace

| **Attributes levels** | **Birthplace:city** | | | | **Birthplace:county or rural** | | | |
| --- | --- | --- | --- | --- | --- | --- | --- | --- |
|  | **β** | **SE** | **SD** | **SE** | **β** | **SE** | **SD** | **SE** |
| **Work location (ref.Township or village)** |  |  |  |  |  |  |  |  |
| County | 0.777^***^ | 0.165 | 0.591^*^ | 0.273 | 0.932^***^ | 0.098 | 0.821^***^ | 0.141 |
| City | 2.769^***^ | 0.220 | 1.146^***^ | 0.187 | 1.540^***^ | 0.118 | 1.375^***^ | 0.134 |
| **Work unit (ref.Private enterprises)** |  |  |  |  |  |  |  |  |
| State-owned enterprises | 0.994^***^ | 0.189 | 0.716^**^ | 0.248 | 0.694^***^ | 0.111 | 0.742^***^ | 0.172 |
| Foreign-funded enterprises | 0.903^***^ | 0.197 | 0.971^***^ | 0.232 | 0.929^***^ | 0.103 | 0.382 | 0.248 |
| Public institutions | 0.924^***^ | 0.190 | 0.525 | 0.355 | 1.022^***^ | 0.116 | 0.928^***^ | 0.145 |
| **Management style (ref.Semi-open management)** |  |  |  |  |  |  |  |  |
| Open management | 0.101 | 0.116 | 0.262 | 0.239 | 0.041 | 0.063 | 0.039 | 0.272 |
| **Training opportunity (ref.Insufficient)** |  |  |  |  |  |  |  |  |
| Average | 0.637^***^ | 0.161 | 0.377 | 0.309 | 0.271^**^ | 0.085 | 0.166 | 0.293 |
| Sufficient | 0.870^***^ | 0.160 | 0.171 | 0.449 | 0.729^***^ | 0.090 | 0.626^***^ | 0.148 |
| **Years to promotion (ref.5years)** |  |  |  |  |  |  |  |  |
| 2years | 1.042^***^ | 0.137 | 0.721^***^ | 0.168 | 0.943^***^ | 0.073 | 0.579^***^ | 0.117 |
| **Monthly income** | 0.000631^***^ | 0.000044 | 0.000161^***^ | 0.000039 | 0.000666^***^ | 0.000030 | 0.000213^***^ | 0.000025 |
| **Opt-out** | 7.520^***^ | 0.554 | 2.222^***^ | 0.253 | 5.241^***^ | 0.290 | 2.221^***^ | 0.200 |
| **Log likelihood** | -1074.231 | | | | -2965.914 | | | |
| **Respondents, n** | 132 | | | | 368 | | | |
| **Observations, n** | 4752 | | | | 13248 | | | |

Note:β-coefficient, SE-standard error, SD-standard deviation, ref-reference, 95% CI = 95% Confidence Interval, ^*^p < 0.05, ^**^p < 0.01, ^***^p < 0.001.

**Supplementary Table 5.** Sub-group analysis: Annual family income

| **Attributes levels** | **Family income≤50000** | | | | **Family income＞50000** | | | |
| --- | --- | --- | --- | --- | --- | --- | --- | --- |
|  | **β** | **SE** | **SD** | **SE** | **β** | **SE** | **SD** | **SE** |
| **Work location (ref.Township or village)** |  |  |  |  |  |  |  |  |
| County | 0.835^***^ | 0.105 | 0.396 | 0.253 | 1.002^***^ | 0.146 | 1.228^***^ | 0.176 |
| City | 1.451^***^ | 0.132 | 1.235^***^ | 0.139 | 2.482^***^ | 0.176 | 1.483^***^ | 0.165 |
| **Work unit (ref.Private enterprises)** |  |  |  |  |  |  |  |  |
| State-owned enterprises | 0.683^***^ | 0.132 | 0.953^***^ | 0.166 | 0.944^***^ | 0.147 | 0.599^*^ | 0.296 |
| Foreign-funded enterprises | 0.829^***^ | 0.120 | 0.354 | 0.236 | 1.112^***^ | 0.149 | 0.875^***^ | 0.205 |
| Public institutions | 0.914^***^ | 0.138 | 1.094^***^ | 0.156 | 1.125^***^ | 0.146 | 0.601^*^ | 0.245 |
| **Management style (ref.Semi-open management)** |  |  |  |  |  |  |  |  |
| Open management | 0.045 | 0.074 | 0.221 | 0.211 | 0.075 | 0.087 | 0.136 | 0.242 |
| **Training opportunity (ref.Insufficient)** |  |  |  |  |  |  |  |  |
| Average | 0.179 | 0.102 | 0.442^*^ | 0.190 | 0.606^***^ | 0.119 | 0.159 | 0.291 |
| Sufficient | 0.718^***^ | 0.103 | 0.554^**^ | 0.180 | 0.864^***^ | 0.121 | 0.409 | 0.216 |
| **Years to promotion (ref.5years)** |  |  |  |  |  |  |  |  |
| 2years | 0.925^***^ | 0.089 | 0.685^***^ | 0.120 | 1.108^***^ | 0.108 | 0.811^***^ | 0.138 |
| **Monthly income** | 0.000643^***^ | 0.000036 | 0.000200^***^ | 0.000026 | 0.000715^***^ | 0.000038 | 0.000209^***^ | 0.000028 |
| **Opt-out** | 4.886^***^ | 0.344 | 2.347^***^ | 0.230 | 7.306^***^ | 0.413 | 2.544^***^ | 0.243 |
| **Log likelihood** | -2153.358 | | | | -1888.915 | | | |
| **Respondents, n** | 263 | | | | 237 | | | |
| **Observations, n** | 9468 | | | | 8532 | | | |

Note:β-coefficient, SE-standard error, SD-standard deviation, ref-reference, 95% CI = 95% Confidence Interval, ^*^p < 0.05, ^**^p < 0.01, ^***^p < 0.001.

**Supplementary Table 6.** Sub-group analysis: Sex

| **Attributes levels** | **Male** | | | | **Female** | | | |
| --- | --- | --- | --- | --- | --- | --- | --- | --- |
|  | **β** | **SE** | **SD** | **SE** | **β** | **SE** | **SD** | **SE** |
| **Work location (ref.Township or village)** |  |  |  |  |  |  |  |  |
| County | 0.785^***^ | 0.160 | 0.735^*^ | 0.306 | 0.948^***^ | 0.100 | 0.760^***^ | 0.138 |
| City | 1.651^***^ | 0.196 | 1.275^***^ | 0.188 | 2.044^***^ | 0.129 | 1.367^***^ | 0.122 |
| **Work unit (ref.Private enterprises)** |  |  |  |  |  |  |  |  |
| State-owned enterprises | 0.684^***^ | 0.192 | 1.109^***^ | 0.264 | 0.822^***^ | 0.112 | 0.660^***^ | 0.186 |
| Foreign-funded enterprises | 0.860^***^ | 0.183 | 0.880^***^ | 0.239 | 0.961^***^ | 0.106 | 0.329 | 0.246 |
| Public institutions | 0.710^***^ | 0.181 | 0.702^**^ | 0.252 | 1.110^***^ | 0.119 | 0.957^***^ | 0.137 |
| **Management style (ref.Semi-open management)** |  |  |  |  |  |  |  |  |
| Open management | 0.338^**^ | 0.108 | 0.030 | 0.315 | -0.054 | 0.065 | 0.155 | 0.172 |
| **Training opportunity (ref.Insufficient)** |  |  |  |  |  |  |  |  |
| Average | 0.373^**^ | 0.142 | 0.023 | 0.261 | 0.367^***^ | 0.091 | 0.430^*^ | 0.202 |
| Sufficient | 0.812^***^ | 0.148 | 0.549^*^ | 0.245 | 0.772^***^ | 0.092 | 0.474^**^ | 0.160 |
| **Years to promotion (ref.5years)** |  |  |  |  |  |  |  |  |
| 2years | 0.901^***^ | 0.127 | 0.636^***^ | 0.156 | 1.026^***^ | 0.079 | 0.705^***^ | 0.104 |
| **Monthly income** | 0.000643^***^ | 0.000047 | 0.000278^***^ | 0.000043 | 0.000671^***^ | 0.000029 | 0.000180^***^ | 0.000020 |
| **Opt-out** | 5.860^***^ | 0.495 | 2.683^***^ | 0.298 | 5.845^***^ | 0.294 | 2.384^***^ | 0.192 |
| **Log likelihood** | -1171.636 | | | | -2890.109 | | | |
| **Respondents, n** | 142 | | | | 358 | | | |
| **Observations, n** | 5112 | | | | 12888 | | | |

Note:β-coefficient, SE-standard error, SD-standard deviation, ref-reference, 95% CI = 95% Confidence Interval, ^*^p < 0.05, ^**^p < 0.01, ^***^p < 0.001.

**Supplementary Table 7.** Sub-group analysis: Single child status

| **Attributes levels** | **Single child** | | | | **Non-single child** | | | |
| --- | --- | --- | --- | --- | --- | --- | --- | --- |
|  | **β** | **SE** | **SD** | **SE** | **β** | **SE** | **SD** | **SE** |
| **Work location (ref.Township or village)** |  |  |  |  |  |  |  |  |
| County | 1.058^***^ | 0.153 | 0.489 | 0.411 | 0.828^***^ | 0.103 | 0.824^***^ | 0.134 |
| City | 2.707^***^ | 0.204 | 1.408^***^ | 0.166 | 1.520^***^ | 0.122 | 1.284^***^ | 0.134 |
| **Work unit (ref.Private enterprises)** |  |  |  |  |  |  |  |  |
| State-owned enterprises | 0.892^***^ | 0.177 | 0.970^***^ | 0.235 | 0.727^***^ | 0.116 | 0.713^***^ | 0.185 |
| Foreign-funded enterprises | 1.027^***^ | 0.175 | 0.995^***^ | 0.219 | 0.884^***^ | 0.107 | 0.167 | 0.259 |
| Public institutions | 0.975^***^ | 0.167 | 0.681^**^ | 0.238 | 1.019^***^ | 0.124 | 0.963^***^ | 0.153 |
| **Management style (ref.Semi-open management)** |  |  |  |  |  |  |  |  |
| Open management | 0.074 | 0.102 | 0.077 | 0.211 | 0.061 | 0.066 | 0.113 | 0.394 |
| **Training opportunity (ref.Insufficient)** |  |  |  |  |  |  |  |  |
| Average | 0.715^***^ | 0.147 | 0.641^**^ | 0.205 | 0.204^*^ | 0.091 | 0.157 | 0.243 |
| Sufficient | 0.904^***^ | 0.149 | 0.636^**^ | 0.233 | 0.725^***^ | 0.095 | 0.520^***^ | 0.152 |
| **Years to promotion (ref.5years)** |  |  |  |  |  |  |  |  |
| 2years | 1.017^***^ | 0.125 | 0.862^***^ | 0.145 | 0.982^***^ | 0.080 | 0.645^***^ | 0.119 |
| **Monthly income** | 0.000741^***^ | 0.000048 | 0.000254^***^ | 0.000038 | 0.000638^***^ | 0.000030 | 0.000187^***^ | 0.000023 |
| **Opt-out** | 7.643^***^ | 0.516 | 2.679^***^ | 0.267 | 5.081^***^ | 0.319 | 2.332^***^ | 0.212 |
| **Log likelihood** | -1469.302 | | | | -2570.426 | | | |
| **Respondents, n** | 185 | | | | 315 | | | |
| **Observations, n** | 6660 | | | | 11340 | | | |

Note:β-coefficient, SE-standard error, SD-standard deviation, ref-reference, 95% CI = 95% Confidence Interval, ^*^p < 0.05, ^**^p < 0.01, ^***^p < 0.001.

**Supplementary Table 8.** Sub-group analysis: Universities

| **Attributes levels** | **East** | | | | **Middle** | | | | **West** | | | |
| --- | --- | --- | --- | --- | --- | --- | --- | --- | --- | --- | --- | --- |
|  | **β** | **SE** | **SD** | **SE** | **β** | **SE** | **SD** | **SE** | **β** | **SE** | **SD** | **SE** |
| **Work location (ref.Township or village)** |  |  |  |  |  |  |  |  |  |  |  |  |
| County | 0.835^***^ | 0.117 | 0.936^***^ | 0.182 | 1.128^***^ | 0.186 | 0.746^**^ | 0.246 | 1.124^***^ | 0.242 | 1.305^***^ | 0.299 |
| City | 2.081^***^ | 0.150 | 1.456^***^ | 0.135 | 1.915^***^ | 0.241 | 1.551^***^ | 0.228 | 2.247^***^ | 0.270 | 1.206^***^ | 0.248 |
| **Work unit (ref.Private enterprises)** |  |  |  |  |  |  |  |  |  |  |  |  |
| State-owned enterprises | 0.856^***^ | 0.138 | 1.056^***^ | 0.173 | 0.523^**^ | 0.201 | 0.622^*^ | 0.292 | 1.135^***^ | 0.266 | 1.313^***^ | 0.300 |
| Foreign-funded enterprises | 1.149^***^ | 0.134 | 0.831^***^ | 0.168 | 0.647^**^ | 0.202 | 0.645^*^ | 0.263 | 1.134^***^ | 0.221 | 0.062 | 0.330 |
| Public institutions | 1.149^***^ | 0.140 | 1.061^***^ | 0.158 | 0.913^***^ | 0.206 | 0.585 | 0.330 | 0.854^**^ | 0.266 | 1.415^***^ | 0.295 |
| **Management style (ref.Semi-open management)** |  |  |  |  |  |  |  |  |  |  |  |  |
| Open management | 0.043 | 0.080 | 0.426^**^ | 0.130 | -0.022 | 0.118 | 0.007 | 0.205 | 0.181 | 0.146 | 0.552^*^ | 0.229 |
| **Training opportunity (ref.Insufficient)** |  |  |  |  |  |  |  |  |  |  |  |  |
| Average | 0.502^***^ | 0.108 | 0.526^**^ | 0.167 | 0.221 | 0.160 | 0.165 | 0.329 | 0.223 | 0.201 | 0.826^**^ | 0.286 |
| Sufficient | 1.016^***^ | 0.113 | 0.733^***^ | 0.172 | 0.359^*^ | 0.161 | 0.080 | 0.276 | 0.834^***^ | 0.209 | 0.913^***^ | 0.273 |
| **Years to promotion (ref.5years)** |  |  |  |  |  |  |  |  |  |  |  |  |
| 2years | 1.204^***^ | 0.094 | 0.601^***^ | 0.127 | 1.072^***^ | 0.168 | 1.058^***^ | 0.220 | 0.594^***^ | 0.634 | 1.581^***^ | 0.286 |
| **Monthly income** | 0.000778^***^ | 0.000038 | 0.000218^***^ | 0.000025 | 0.000637^***^ | 0.000057 | 0.000243^***^ | 0.000043 | 0.000674^***^ | 0.000066 | 0.000178^***^ | 0.000037 |
| **Opt-out** | 7.043^***^ | 0.374 | 2.761^***^ | 0.224 | 4.679^***^ | 0.532 | 2.402^***^ | 0.339 | 6.368^***^ | 0.160 | 0.771^***^ | 0.202 |
| **Log likelihood** | -2420.826 | | | | -830.725 | | | | -775.799 | | | |
| **Respondents, n** | 309 | | | | 102 | | | | 89 | | | |
| **Observations, n** | 11124 | | | | 3672 | | | | 3204 | | | |

Note:β-coefficient, SE-standard error, SD-standard deviation, ref-reference, 95% CI = 95% Confidence Interval, ^*^p < 0.05, ^**^p < 0.01, ^***^p < 0.001.
